# Supplementary material for: Web use remains highly regional even in the age of global platform monopolies
Source: PLoS One. 2023 Jan 11;18(1):e0278594. doi: 10.1371/journal.pone.0278594 (PMC9833580; doi:10.1371/journal.pone.0278594)
Supplement: S1 Table — (DOCX) [file pone.0278594.s001.docx]

| **S1 Table. Scales of data collection***.* | | |
| --- | --- | --- |
| **Platforms** |  | **Countries** |
| **Alexa**  September:  62,000 websites (29,272 unique)  November:  62,000 websites (29,444 unique) | | Albania, Algeria, Angola, Argentina, Armenia, Australia, Austria, Azerbaijan, Bahrain, Bangladesh, Belarus, Belgium, Bolivia, Bosnia and Herzegovina, Brazil, Brunei, Bulgaria, Cambodia, Cameroon, Canada, Chile, China, Colombia, Costa Rica, Croatia, Czech Republic, Denmark, Dominican Republic, Ecuador, Egypt, El Salvador, Estonia, Ethiopia, Finland, France, Georgia, Germany, Ghana, Greece, Honduras, Hong Kong, Hungary, India, Indonesia, Iran, Iraq, Ireland, Israel, Italy, Jamaica, Japan, Jordan, Kazakhstan, Kenya, Kuwait, Latvia, Lebanon, Libya, Lithuania, Macedonia, Madagascar, Malaysia, Mauritania, Mauritius, Mexico, Moldova, Mongolia, Morocco, Myanmar, Nepal, Netherlands, New Zealand, Nicaragua, Nigeria, Norway, Oman, Pakistan, Palestinian Territory, Panama, Paraguay, Peru, Philippines, Poland, Portugal, Puerto Rico, Qatar, Romania, Russia, Rwanda, Saudi Arabia, Senegal, Serbia, Singapore, Slovakia, Slovenia, Somalia, South Africa, South Korea, Spain, Sri Lanka, Sudan, Sweden, Switzerland, Syrian Arab Republic, Taiwan, Tajikistan, Tanzania, Thailand, Trinidad and Tobago, Tunisia, Turkey, Turkmenistan, Uganda, Ukraine, United Arab Emirates, United Kingdom, United States, Uruguay, Uzbekistan, Venezuela, Vietnam, Yemen, Zambia, and Zimbabwe (124 countries) |
| **YouTube**  September:  3,189,397 videos (54,777 unique)  November:  1,967,331 videos (58,442 unique) | | Algeria, Argentina, Australia, Austria, Azerbaijan, Bahrain, Belarus, Belgium, Bolivia, Bosnia and Herzegovina, Brazil, Bulgaria, Canada, Chile, Colombia, Costa Rica, Croatia, Cyprus, Czech Republic, Denmark, Dominican Republic, Ecuador, El Salvador, Estonia, Finland, France, Georgia, Germany, Ghana, Greece, Honduras, Hong Kong, Hungary, Iceland, India, Indonesia, Iraq, Ireland, Israel, Italy, Jamaica, Japan, Jordan, Kazakhstan, Kenya, Kuwait, Latvia, Lebanon, Lithuania, Luxembourg, Macedonia, Malaysia, Malta, Mexico, Morocco, Nepal, Netherlands, New Zealand, Nicaragua, Nigeria, Norway, Oman, Pakistan, Panama, Paraguay, Peru, Philippines, Poland, Portugal, Puerto Rico, Qatar, Romania, Russia, Saudi Arabia, Senegal, Serbia, Singapore, Slovakia, Slovenia, South Africa, South Korea, Spain, Sri Lanka, Sweden, Switzerland, Taiwan, Tanzania, Thailand, Tunisia, Turkey, Uganda, Ukraine, United Arab Emirates, United Kingdom, United States, Uruguay, Vietnam, and Zimbabwe (98 countries) |
| **Twitter**  September:  2,007,995 topics (124,390 unique)  November:  1,576,305 topics (104,909 unique) | | Algeria, Argentina, Australia, Austria, Bahrain, Belarus, Belgium, Brazil, Canada, Chile, Colombia, Denmark, Dominican Republic, Ecuador, Egypt, France, Germany, Ghana, Greece, India, Indonesia, Ireland, Israel, Italy, Japan, Jordan, Kenya, Kuwait, Latvia, Lebanon, Malaysia, Mexico, Netherlands, New Zealand, Nigeria, Norway, Oman, Pakistan, Panama, Peru, Philippines, Poland, Portugal, Puerto Rico, Qatar, Russia, Saudi Arabia, Singapore, South Africa, South Korea, Spain, Sweden, Switzerland, Thailand, Turkey, Ukraine, United Arab Emirates, United Kingdom, United States, Venezuela, and Vietnam (61 countries) |
