# Supplementary material for: Web use remains highly regional even in the age of global platform monopolies
Source: PLoS One. 2023 Jan 11;18(1):e0278594. doi: 10.1371/journal.pone.0278594 (PMC9833580; doi:10.1371/journal.pone.0278594)
Supplement: S2 Table — (Countries with at least one non-zero betweenness, in log scale, sorted by Twitter). (DOCX) [file pone.0278594.s002.docx]

**S2 Table. Network betweenness centralities of the first month for Website traffic (Alexa), YouTube, and Twitter. (Countries with at least one non-zero betweenness, in log scale, sorted by Twitter).**

| **Countries** | **Alexa** | **YouTube** | **Twitter** |  | **Countries** | **Alexa** | | **YouTube** | **Twitter** |
| --- | --- | --- | --- | --- | --- | --- | --- | --- | --- |
| Australia | 0 | 207 | 10 |  | New Zealand | 0 | | 24 | 12 |
| Austria | 0 | 39 | 0 |  | Nigeria | 0 | | 57 | 174 |
| Bahrain | 0 | 8 | 23 |  | Norway | 0 | | 76 | 2 |
| Belarus | 0 | 50 | 2 |  | Oman | 0 | | 21 | 0 |
| Belgium | 0 | 83 | 103 |  | Pakistan | 0 | | 0 | 99 |
| Canada | 0 | 1 | 139 |  | Panama | 0 | | 88 | 0 |
| Chile | 0 | 67 | 1 |  | Peru | 0 | | 116 | 33 |
| Colombia | 0 | 0 | 31 |  | Philippines | 0 | | 0 | 58 |
| Denmark | 0 | 11 | 2 |  | Portugal | | 0 | 121 | 0 |
| Dominican Republic | 0 | 0 | 58 |  | Puerto Rico | | 0 | 57 | 22 |
| Ecuador | 0 | 0 | 3 |  | Qatar | | 0 | 0 | 79 |
| France | 0 | 0 | 28 |  | Russia | | 0 | 0 | 15 |
| Germany | 0 | 0 | 15 |  | Saudi Arabia | | 0 | 7 | 0 |
| Greece | 0 | 77 | 0 |  | Singapore | | 9 | 162 | 405 |
| Guatemala | 0 | 0 | 31 |  | South Africa | | 0 | 508 | 0 |
| Indonesia | 0 | 94 | 0 |  | Spain | | 0 | 45 | 4 |
| Ireland | 0 | 15 | 9 |  | Sweden | | 0 | 0 | 4 |
| Kuwait | 0 | 7 | 57 |  | Switzerland | | 0 | 126 | 105 |
| Latvia | 0 | 161 | 0 |  | Ukraine | | 0 | 0 | 2 |
| Lebanon | 0 | 0 | 11 |  | United Arab Emirates | | 0 | 179 | 317 |
| Malaysia | 0 | 46 | 168 |  | United Kingdom | | 0 | 2 | 23 |
| Mexico | 0 | 51 | 121 |  | United States | | 0 | 1 | 58 |
| Netherlands | 3 | 0 | 0 |  |  | |  |  |  |
