# Supplementary material for: Web use remains highly regional even in the age of global platform monopolies
Source: PLoS One. 2023 Jan 11;18(1):e0278594. doi: 10.1371/journal.pone.0278594 (PMC9833580; doi:10.1371/journal.pone.0278594)
Supplement: S3 Table — (DOCX) [file pone.0278594.s003.docx]

| **S3 Table. Descriptive statistics for matrices of Alexa, YouTube, and Twitter in September and November** | | | | | | | | | | | |
| --- | --- | --- | --- | --- | --- | --- | --- | --- | --- | --- | --- |
| **With all available countries** | | | | | |  |  | **With 59 common countries*** | | | |
| **September** | | | | | | | | | | | |
|  | Weighted degrees | | |  | Proportion of nonzero  betweenness centralities | |  |  | Weighted degrees | | |
|  | *Mean* | *S.D.* | *Gini* | *(#of clusters)* |  |  |  |  | *Mean* | *S.D.* | *Gini* |
| Alexa (124 countries) | 39.11 | 6.25 | .08 | (3) | .03 | |  | Alexa | 20.74 | 2.92 | .07 |
| YouTube (98 countries) | 7.19 | 3.22 | .25 | (4) | .55 | |  | YouTube | 4.11 | 2.10 | .29 |
| Twitter (61 countries) | 1.54 | 0.70 | .25 | (5) | .56 | |  | Twitter | 1.45 | 0.66 | .25 |
| **November** |  |  |  |  |  | |  |  |  |  |  |
| Alexa (124 countries) | 39.98 | 6.31 | .08 | (3) | .04 | |  | Alexa | 20.43 | 2.89 | .07 |
| YouTube (98 countries) | 5.84 | 3.07 | .30 | (3) | .60 | |  | YouTube | 3.98 | 2.00 | .29 |
| Twitter (61 countries) | 1.20 | 0.49 | .23 | (5) | .57 | |  | Twitter | 1.42 | 0.65 | .25 |

*Common countries among three platforms include Algeria, Argentina, Australia, Austria, Bahrain, Belarus, Belgium, Brazil, Canada, Chile, Colombia, Denmark, Dominican Republic, Ecuador, France, Germany, Ghana, Greece, India, Indonesia, Ireland, Israel, Italy, Japan, Jordan, Kenya, Kuwait, Latvia, Lebanon, Malaysia, Mexico, Netherlands, New Zealand, Nigeria, Norway, Oman, Pakistan, Panama, Peru, Philippines, Poland, Portugal, Puerto Rico, Qatar, Russia, Saudi Arabia, Singapore, South Africa, South Korea, Spain, Sweden, Switzerland, Thailand, Turkey, Ukraine, United Arab Emirates, United Kingdom, United States, and Vietnam (59).
