# Supplementary material for: Web use remains highly regional even in the age of global platform monopolies
Source: PLoS One. 2023 Jan 11;18(1):e0278594. doi: 10.1371/journal.pone.0278594 (PMC9833580; doi:10.1371/journal.pone.0278594)
Supplement: S4 Table — (DOCX) [file pone.0278594.s004.docx]

| **S4 Table. QAP correlations across variable matrices (based on 59 common countries).** | | | | | | | |  |  |  |
| --- | --- | --- | --- | --- | --- | --- | --- | --- | --- | --- |
|  | 1 | 2 | 3 | 4 | 5 | 6 | 7 | 8 | 9 |  |
| 1. Alexa similarity |  |  |  |  |  |  |  |  |  |  |
| 1. YouTube similarity | .44^***^ |  |  |  |  |  |  |  |  |  |
| 1. Twitter topic similarity | .32^***^ | .47^***^ |  |  |  |  |  |  |  |  |
| 1. Language composition | .49^***^ | .70^***^ | .47^***^ |  |  |  |  |  |  |  |
| 1. Sharing border | .18^***^ | .25^***^ | .16^***^ | .23^***^ |  |  |  |  |  |  |
| 1. Internet market size | −.01 | −.14^**^ | −.05 | −.02 | .01 |  |  |  |  |  |
| 1. US effect | .13^*^ | .004 | .03 | .14^*^ | −.01 | .64^*^ |  |  |  |  |
| 1. China effect^#^ | −.45^**^ | NA | NA | −.05 | .06^**^ | .78^**^ | −.01 |  |  |  |
| 1. English prevalence | .11^*^ | .04 | .26^***^ | .17^***^ | .02 | .27^*^ | .46^*^ | −.01^#^ |  |  |
| ^*^ p < .05 ^**^ p < .01 ^***^ p < .001  # Correlations of China effect are based all available countries, except for YouTube and Twitter (did not have data for China). | | | | | | | | | | |
