# Supplementary material for: Web use remains highly regional even in the age of global platform monopolies
Source: PLoS One. 2023 Jan 11;18(1):e0278594. doi: 10.1371/journal.pone.0278594 (PMC9833580; doi:10.1371/journal.pone.0278594)
Supplement: S5 Table — A. QAP regressions for website traffic (Alexa) similarity across countries (September, N = 124). B. QAP regressions for website traffic (Alexa) similarity across countries (November, N = 124). (DOCX) [file pone.0278594.s005.docx]

| **S5A Table. QAP regressions for website traffic (Alexa) similarity across countries (September, N = 124).** | | | |
| --- | --- | --- | --- |
| Variables | *b* | | |
|  | Block 1 | Block 2 | Block 3 |
| Intercept | 0.31^***^ | 0.31^***^ | 0.31^***^ |
| Language composition | .04^***^ | .03^***^ | .03^***^ |
| Sharing border | .02^*^ | .02^**^ | .02^**^ |
| Internet market size | −.02^**^ | −.004 | −.004 |
| US effect |  | .04 | −.04 |
| China effect |  | −.25^**^ | −.25^***^ |
| English prevalence |  |  | .002 |
| R^2^ | .32^***^ | .39^***^ | .39^***^ |
| Adjusted R^2^ | .32^***^ | .39^***^ | .39^***^ |
| Notes: 1,000 permutations for estimating standard errors.  Coefficients presented are standardized coefficients.  ^*^ p < .05 ^**^ p < .01 ^***^ p < .001 | | | |

| **S5B Table. QAP regressions for website traffic (Alexa) similarity across countries (November, N = 124).** | | | |
| --- | --- | --- | --- |
| Variables | *b* | | |
|  | Block 1 | Block 2 | Block 3 |
| Intercept | 0.32^***^ | 0.32^***^ | 0.32^***^ |
| Language composition | .03^***^ | .03^***^ | .03^***^ |
| Sharing border | .02^*^ | .02^**^ | .02^**^ |
| Internet market size | −.02^**^ | −.005 | −.005 |
| US effect |  | .04 | −.04 |
| China effect |  | −.26^**^ | −.26^***^ |
| English prevalence |  |  | .003 |
| R^2^ | .31^***^ | .39^***^ | .39^***^ |
| Adjusted R^2^ | .31^***^ | .39^***^ | .39^***^ |
| Notes: 1,000 permutations for estimating standard errors.  Coefficients presented are standardized coefficients.  ^*^ p < .05 ^**^ p < .01 ^***^ p < .001 | | | |
