# Supplementary material for: Web use remains highly regional even in the age of global platform monopolies
Source: PLoS One. 2023 Jan 11;18(1):e0278594. doi: 10.1371/journal.pone.0278594 (PMC9833580; doi:10.1371/journal.pone.0278594)
Supplement: S6 Table — A. QAP regressions for YouTube trending video similarity across countries (September, N = 98). B. QAP regressions for YouTube trending video similarity across countries (November, N = 98). (DOCX) [file pone.0278594.s006.docx]

| **S6A Table. QAP regressions for YouTube trending video similarity across countries (September, N = 98).** | | | |
| --- | --- | --- | --- |
| Variables | *b* | | |
|  | Block 1 | Block 2 | Block 3 |
| Intercept | 0.06^***^ | 0.06^**^ | 0.06^**^ |
| Language composition | .07^***^ | .07^***^ | .07^***^ |
| Sharing border | .08^***^ | .08^***^ | .08^***^ |
| Internet market size | −.02^***^ | −.02^***^ | −.02^***^ |
| US effect |  | .01 | .02 |
| English prevalence |  |  | −.005^**^ |
| R^2^ | .50^***^ | .50^***^ | .50^***^ |
| Adjusted R^2^ | .50^***^ | .50^***^ | .50^***^ |
| Notes: 1,000 permutations for estimating standard errors.  Coefficients presented are standardized coefficients.  ^*^ p < .05 ^**^ p < .01 ^***^ p < .001 | | | |

| **S6B Table. QAP regressions for YouTube trending video similarity across countries (November, N = 98).** | | | |
| --- | --- | --- | --- |
| Variables | *b* | | |
|  | Block 1 | Block 2 | Block 3 |
| Intercept | 0.05^**^ | 0.05^*^ | 0.05^*^ |
| Language composition | .06^***^ | .06^***^ | .06^***^ |
| Sharing border | .09^***^ | .09^***^ | .09^***^ |
| Internet market size | −.02^***^ | −.03^***^ | −.03^***^ |
| US effect |  | .03 | .04 |
| English prevalence |  |  | −.004^*^ |
| R^2^ | .47^***^ | .47^***^ | .47^***^ |
| Adjusted R^2^ | .47^***^ | .47^***^ | .47^***^ |
| Notes: 1,000 permutations for estimating standard errors.  Coefficients presented are standardized coefficients.  ^*^ p < .05 ^**^ p < .01 ^***^ p < .001 | | | |
