# Supplementary material for: Web use remains highly regional even in the age of global platform monopolies
Source: PLoS One. 2023 Jan 11;18(1):e0278594. doi: 10.1371/journal.pone.0278594 (PMC9833580; doi:10.1371/journal.pone.0278594)
Supplement: S7 Table — A. QAP regressions for Twitter trending topic similarity across countries (September, N = 61). B. QAP regressions for Twitter trending topic similarity across countries (November, N = 61). (DOCX) [file pone.0278594.s007.docx]

| **S7A Table. QAP regressions for Twitter trending topic similarity across countries (September, N = 61).** | | | | |
| --- | --- | --- | --- | --- |
| Variables | *b* | | | |
|  | Block 1 | Block 2 | Block 3 | |
| Intercept | 0.02^**^ | 0.02^**^ | | 0.02^**^ |
| Language composition | .01^***^ | .01^***^ | | .01^***^ |
| Sharing border | .01^*^ | .01^*^ | | .01^**^ |
| Internet market size | −.002 | −.002 | | −.002 |
| US effect |  | −.002 | | −.02 |
| English prevalence |  |  | | .005^***^ |
| R^2^ | .24^***^ | .24^***^ | | .28^***^ |
| Adjusted R^2^ | .24^***^ | .24^***^ | | .28^***^ |
| Notes: 1,000 permutations for estimating standard errors.  Coefficients presented are standardized coefficients.  ^*^ p < .05 ^**^ p < .01 ^***^ p < .001 | | | | |

| **S7B Table. QAP regressions for Twitter trending topic similarity across countries (November, N = 61).** | | | | |
| --- | --- | --- | --- | --- |
| Variables | *b* | | | |
|  | Block 1 | Block 2 | Block 3 | |
| Intercept | 0.02^**^ | 0.02^**^ | | 0.02^**^ |
| Language composition | .01^***^ | .01^***^ | | .01^***^ |
| Sharing border | .02^***^ | .02^***^ | | .02^***^ |
| Internet market size | −.002 | −.001 | | −.001 |
| US effect |  | −.009 | | −.02 |
| English prevalence |  |  | | .002^***^ |
| R^2^ | .25^***^ | .25^***^ | | .26^***^ |
| Adjusted R^2^ | .24^***^ | .25^***^ | | .26^***^ |
| Notes: 1,000 permutations for estimating standard errors.  Coefficients presented are standardized coefficients.  ^*^ p < .05 ^**^ p < .01 ^***^ p < .001 | | | | |
