# Supplementary material for: Web use remains highly regional even in the age of global platform monopolies
Source: PLoS One. 2023 Jan 11;18(1):e0278594. doi: 10.1371/journal.pone.0278594 (PMC9833580; doi:10.1371/journal.pone.0278594)
Supplement: Attachment — Submitted filename: 02_Letter to the Editor.docx [file pone.0278594.s008.docx]

Dear Dr. Bovet and Esteemed Reviewers,

Many thanks for getting our manuscript reviewed and giving us the opportunity to improve the manuscript based on your considered summary of the four detailed and insightful reviews. In this letter, we outline the changes we have made. As per the editor’s suggestions we have been mindful of the collective insights of the reviewers and have followed editorial directions based on the editor’s summary of the two reviewers’ comments. Additionally, we have incorporated some other suggestions made by the reviewers.

We have retained the editor’s comments in this letter and have provided our response point wise below each comment (original comments are indented and bold for clarity).

**Please clearly explain how you performed the clustering of the networks.**

We used a modularity-based Louvain algorithm accords with the overall centrality scores.

**Also, modularity values should not be used to compare "how modular" different networks are. It can only be used to compare the modularity of different clustering of the same network. Indeed, even a random graph (i.e. a graph without community structure) can have a partition with a modularity close to 1. Moreover modularity values depend on the size of the networks. Please remove the modularity values from all the tables comparing different networks and the corresponding discussions. You can leave the number of clusters.**

We appreciate this insight and have now removed modularity values. Also following the editor’s suggestion, we have retained the number of clusters.

Reviewer 1 very helpfully classified their comments into two categories, and we have attempted to respond to each of them.

**(1) missing citations from the literature review**

We apologize for missing some very important related work and now in the revised version cite these on p. 3.

**(2) the need for a complete network analysis, including justification of the use of the chosen centrality measure and network graphics.**

We have added details regarding the network analysis in the method (p. 6) and results (p. 9).

a) In the methods section:

**P 10. L115-124. When reading this, I questioned the use of Twitter. It is primarily an English language site. However, the results justify its inclusion. But it should be discussed here that since it is primarily an English language site, that you expect those results.**

Thanks for Reviewer 1’s suggestion. We have now added the following justification “language could play a smaller role in explaining the usage of Twitter, given the dominance English has on the platform.” on page 4.

**P13. L199. What measure of centrality did you use? Average weighted degree score? Why did you not use eigenvalue or closeness ? Eigenvalue centrality is typically considered a better measure of overall centrality. Here you should explain betweenness in detail, since you rely on its measure to draw your conclusions.**

We used weighted degrees as the measure of centrality as it lends itself to a simple yet elegant interpretation - the higher the weighted degree of a country pair the more similar the two countries are in their web usage. We have no problems calculating closeness and eigenvector scores but given that these networks are projections of bipartite data (websites and countries), interpreting other measures in theoretically meaningful ways is harder. This is compounded by the fact that the tie strengths here indicate similarities rather than distances.

    We now explain some examples related to our betweenness scores to further aid interpretation (on p. 8).

**P14. L250. Perhaps, you should have added the physical distances between capitals or largest. cities. This would account for islands and nearby countries that don't share a border and the variance in distances among countries that don’t share common borders.**

We did not use physical distances as recent studies of internet use on similar scales (e.g., [14]) have shown the usefulness of shared borders over physical distance as a measure.

b) In the results section:

**P15. What are the densities of the three networks? This would help network scholars understand the results. Also, I would include the network graphics with the countries labelled for all three networks. It would make the results much easier to understand. We could then see the various communities of nations and those that occupy the betweenness positions. In particular, as Barnett & Benefield show, these communities are typically formed by common language and geographical proximity. These would help support your argument.**

We did not report densities as for completely connected graphs, which ours were by virtue of being one mode projections of bipartite data, the density is always one and the average of the weighted degree is in effect a measure of its density - we now note that detail in the manuscript (on p. 6).

**P15. L286. You haven't introduced the notion of separate communities of nations. You should and then discuss the meaning of modularity. Note that Barnett & Benefield identify communities of nations based on Facebook ties. Further, this would help explain the results displayed in Figure 1. For example, Singapore connects China, Europe, U.S, India and other English-speaking countries. Canada bridges Chinese, French and English-speaking communities. These communities of nations could then be colored differently in the recommended network graphics.**

We also have now added visualizations of the three graphs for September 2021, the main analysis month which as the reviewer requested also shows the community membership based on communities of nations and as per the very helpful suggestion of Reviewer 1 are colored differently by community membership (see Figure 2 on p.11).

We also thank reviewer 1 for pointing out some grammatical mistakes and we fixed them accordingly.

Reviewer #2 had the following concerns,

**Comparability of the three data sources given the very different geographical coverage. The number of countries in the Alexa-, YouTube- and Twitter-based data is very different. They represent three distinct clusters of the global internet, but the analysis does not really take this into account and compares the three databases on a one-to-one basis. (E.g., in Table 1. The trends for Alexa, YouTube and Twitter are important, but directly comparing the indicators might be misleading because they refer to very different geographical regions of the world.) I suggest the authors to run the analysis only on countries that are included in all three data sources and report the results (at least in the supplementary material).**

**Without this step, I think conclusions such as "it appears that consumption of content on social media platforms could overall be more homogeneous globally than website traffic" could be somewhat misleading. This sentence implies that global website traffic is compared to the same global social media platform use, but this is not true because of the very different country coverage of the two sources.**

In the original MS, we had additional analysis of all the 59 common countries among those three platforms in Table S3. However, since Reviewer 2 notes and we agree that this detail is important we have now incorporated this analysis in the main text Table 1 on p. 8.

Some other minor questions:

**How accurate is data from Alexa for the individual countries? Are there any risks that accuracy and valilidity is not the same across countries? (e.g. most of the Alexa toolbar installations were coming from the US, and in several major internet user countries it was much less which might led to certain biases.) This should be briefly touched in the text.**

Previous study has compared Alexa’s U.S. list with ComScore’s 100 most popular sites in the United States and found a high correlation (ρ = .69). But we understand that not every single Internet user that installs and uses the toolbar, most people tend to not weigh Alexa Rank very heavily. Alexa itself even admits that “Alexa's traffic estimates and ranks are based on the browsing behavior of people in our global data panel which is a sample of all internet users.” No traffic estimator (e.g., SimilarWeb or ComScore) will be perfect. The data Alexa provides can help you in most cases to have a “general” idea of how popular a website is, hence, the reason we still considered useful.

**Where is the data for Ethnology coming from? Probably there is no better source for language data, but I did not find any information about the methodology behind this database. (Again, this is a question regarding the accuracy of the datasets.)**

Ethnologue is currently the most comprehensive catalog of the known spoken languages globally. Ethnologue is built by SIL International, the leader in field linguistics. They have thousands of staff all over the world. Ethnologue data is sourced from a number of places. They include staff directly (language survey, direct experience), government census data, reputable online sources, and relationships with local language speakers or other linguists. The database provides estimated numbers of total language speakers for all languages spoken by more than 1% of a country’s population (see also Ronen et al., 2014, for a study using this source). The existence of estimate for every known language in every country provides a useful resource for the assessment of language vitality globally, regionally, and country-by-country. Details of data collection and methodology can be found here <https://www.ethnologue.com/about/language-info> and <https://www.ethnologue.com/about/language-status>. We also cross-checked Ethnologue data with other updated sources, such as Central Intelligence Agency (CIA) World Factbook which gave us confidence on the accuracy of the data.

**Why do the numbers for Youtube differ so much between September and November? Are there any technical or other reasons for this?**

While we aimed to collect the first 200 trending videos for 98 countries, four times per day, YouTube did not consistently return 200 videos for some countries for each data collection. The actual number of trending videos returned from API did fluctuate. For example, for Uganda, we were able to collect 411 trending videos on September 16, 2019, but only 206 videos on November 4, 2019, within our four-time daily collection. However, since our analysis eventually focused on creating a monthly ranking list for each country independently, the difference in the number of videos collected between these two months would pose minimal problems in our analysis.

**Regarding the journal requirements:**

- The authors received no specific funding for this work.
- Data Availability we wish to make changes to our Data Availability statement. We will comply with the Minimal datset policy of the journal
